# Promoted CD4^+^ T cell-derived IFN-γ/IL-10 by photobiomodulation therapy modulates neurogenesis to ameliorate cognitive deficits in APP/PS1 and 3xTg-AD mice

**DOI:** 10.1186/s12974-022-02617-5

**Published:** 2022-10-10

**Authors:** Xiaolei Wu, Qi Shen, Haocai Chang, Junyu Li, Da Xing

**Affiliations:** 1 MOE Key Laboratory of Laser Life Science and Institute of Laser Life Science, College of Biophotonics, South China Normal University, Guangzhou, 510631 China; 2 Guangdong Provincial Key Laboratory of Laser Life Science, College of Biophotonics, South China Normal University, Guangzhou, 510631 China

**Keywords:** Alzheimer's disease, NSCs, Neurogenesis, Cognitive deficits, IFN-γ/IL-10, CD4^+^ T cell, JAK2/STAT4/STAT5 pathway

## Abstract

**Background:**

The immune system has been implicated in synaptic plasticity, inflammation, and the progression of Alzheimer's disease (AD). However, there were few studies on improving the niche microenvironment of neural stem cells (NSCs) in the brain of AD to promote adult hippocampal neurogenesis (AHN) by regulating the function of non-parenchymal immune cells.

**Methods:**

The lymph nodes of amyloid precursor protein/presenilin 1 (APP/PS1) and 3xTg (APP/PS1/tau) mouse models of AD were treated with photobiomodulation therapy (PBMT) for 10 J/cm^2^ per day for 1 month (10 min for each day), T lymphocytes isolated from these two AD models were treated with PBMT for 2 J/cm^2^ (5 min for each time). The NSCs isolated from hippocampus of these two AD models at E14, and the cells were co-cultivated with PBMT-treated T lymphocyte conditioned medium for NSCs differentiation.

**Results:**

Our results showed that PBMT treatment could promote AHN and reverse cognitive deficits in AD mouse model. The expression of interferon-γ (IFN-γ) and interleukin-10 (IL-10) was upregulated in the brain of these two AD models after PBMT treated, which was induced by the activation of Janus kinase 2 (JAK2)-mediated signal transducer and activator of transcription 4 (STAT4)/STAT5 signaling pathway in CD4^+^ T cells. In addition, elevated CD4^+^ T cell levels and upregulated transforming growth factor-β1 (TGFβ1)/insulin-like growth factors-1 (IGF-1)/brain-derived neurotrophic factor (BDNF) protein expression levels were also detected in the brain. More importantly, co-cultivated the PBMT-treated T lymphocyte conditioned medium with NSCs derived from these two AD models was shown to promote NSCs differentiation, which was reflected in the upregulation of both neuronal class-III β-tubulin (Tuj1) and postsynaptic density protein 95 (PSD95), but the effects of PBMT was blocked by reactive oxygen species (ROS) scavenger or JAK2 inhibitor.

**Conclusion:**

Our research suggests that PBMT exerts a beneficial neurogenesis modulatory effect through activating the JAK2/STAT4/STAT5 signaling pathway to promote the expression of IFN-γ/IL-10 in non-parenchymal CD4^+^ T cells, induction of improvement of brain microenvironmental conditions and alleviation of cognitive deficits in APP/PS1 and 3xTg-AD mouse models.

**Supplementary Information:**

The online version contains supplementary material available at 10.1186/s12974-022-02617-5.

## Introduction

Macrophages and dendritic cells, as innate immune cells in the host immune system, mainly phagocytose and present foreign substances bound to major histocompatibility complex (MHC) I and II molecules to T cells, which are then activated to function as effectors [1]. For a long time in the past, the central nervous system (CNS) has always been regarded as a site with relative immune privilege from the immunological perspective due to the existence of blood–brain barrier (BBB) [2]. However, the existence of the meningeal lymphatic system, monocytes, and other immune cells in the CNS led to a gradual evolution in the immunological perspective of brain [3]. These observations have made the research on the CNS immune system not only focus on the function of microglia, but also demonstrated that the immune system plays a crucial role in both physiologic and pathologic brain functions. Alzheimer's disease (AD) is a neurodegenerative disease [4] characterized by amyloid-beta (Aβ) plaque deposition, neurofibrillary tangles (NFTs), reactive gliosis, neuron loss [5–9], accompanied by cerebrovascular amyloidosis, major synaptic changes and cognitive deficits [10–12]. More and more evidence has shown that the AD pathogenesis is not only limited to the neural compartment, but also involves immunological mechanisms [13]. CD4^+^ T cells may play a role in AD, being present in patients’ samples and functionally active in AD mouse models [2, 14–17]. The choroid plexus (CP) of brain, the epithelial layer that forms the blood–cerebrospinal fluid barrier, is a selective gateway for leukocyte to enter the CNS [14], enhancing recruitment monocyte-derived macrophages and T cells in neurodegenerative diseases [18, 19]. Meanwhile, enhanced peripheral CD4^+^ T cells response to Aβ was observed in the brain of AD patients [20, 21], and the magnitude of CD4^+^ T cells is critically controlled by Tregs [15]. In short, the verification of meningeal lymphatic vessels and lymphocytes in the brain indicates that there is a correlation between the CNS and the peripheral immune system, and systemic immune activated immune-dependent cascade may play a beneficial role in the AD pathogenesis.

Neural stem cells (NSCs) are present in the hippocampus and can continuously generate new neurons throughout life in a process called adult hippocampal neurogenesis (AHN), however, the hippocampus, one of the earliest brain regions to be affected in AD [22]. With the deterioration of the brain microenvironment, AHN is damaged, which increases the possibility of reducing the memory impairment and cognitive dysfunction caused by AHN in the AD mouse model [23]. Microglia plays an essential role in maintaining the neural environment, sculpting postnatal neural circuits, promoting neurogenesis, and contributing to NSCs proliferation and differentiation [24]. Importantly, T lymphocytes, which are present in meningeal lymphatics, play a role in neurogenesis and regulate microglial function by releasing soluble factors [25]. CP macrophages survey cerebrospinal fluid (CSF) production and establish relationships with perivascular immune cells to maintain homeostasis [26, 27]. Peripheral immune cells are recruited into the CNS under the influence of brain microenvironment [25], and immune cells can indirectly regulate AHN by releasing cytokines in neurodegenerative diseases [28]. So, is it possible to restrain the reduction of AHN in neurodegenerative diseases by regulating the function of peripheral immune cells and utilizing the process of CNS recruitment of immune cells? In particular, mice with lymphocyte (T and B cell) deficiency showed impaired memory, but the presence of T cell in the meningeal space increased with improved cognitive ability [29]. Recent works have shown that including interleukin-10 (IL-10) and interferon-γ (IFN-γ)-producing T cells positively regulate the learning, memory and behaviors in mice [30, 31]. T cells-deficient nude mice showed AHN impairment [28]. Moreover, CD4^+^ T cells-derived cytokines, IFN-γ, affected the differentiation direction of NSCs by regulating the activity of microglia [32]. These results indicated that T cells affected AHN by interacting with microglia or secreting soluble factors. Specifically, activated microglia locally produced cytokines such as transforming growth factor-β (TGFβ), insulin-like growth factors-1 (IGF-1), and brain-derived neurotrophic factor (BDNF), these cytokines improved the brain microenvironment and positively affected the AHN, however, in the absence of functional CNS-specific T cells, the expression of these cytokines reduced, correspondingly, the AHN was also reduced [25, 28], it was likely that T cells might directly or indirectly affected the function of the NSCs niche to regulate AHN even under the condition of AD pathology.

Recent studies have shown that PBMT, as a non-pharmacologic, non-invasive physiotherapy strategy, could effectively promote nerve regeneration [33, 34], rescued the pathological symptoms of AD [35, 36], and had an effective neuroprotection on the CNS [37, 38]. In addition, PBMT was also being applied to dermatology, dentistry, and immunology [39]. However, there were few studies on improving the niche microenvironment of NSCs in the brain of AD mice to promote AHN by regulating the function of non-parenchymal immune cells. PBMT can activate mitochondrial cytochrome C oxidase (CcO) [40] to cause a transient increase of reactive oxygen species (ROS) in the cytoplasm [41], which act as a natural intracellular messenger, may regulate the expression of downstream genes to regulate the cell activity and physiological functions [42] by activating Janus Kinase (JAK)/ signal transducer and activator of transcriptions (STATs) signaling pathway [43, 44]. In this study, we found that the levels of IFN-γ/IL-10 protein in the brain tissue and serum of the amyloid precursor protein/presenilin1 (APP/PS1) and 3xTg (APP/PS1/tau)-AD transgenic mice were significantly increased after treated the lymph nodes with PBMT for one month. Moreover, the levels of BDNF, IGF-1, and TGFβ1 cytokines in the niches of NSCs were remarkably increased, leading to the promotion of AHN and the alleviation of cognitive deficits symptoms in AD mice. We also found that the molecular mechanism of PBMT upregulating the expression of IFN-γ/IL-10 in CD4^+^ T lymphocytes was mediated by ROS-activated JAK2/STAT4/STAT5 signaling pathway. Collectively, our results demonstrate for the first time that activation of JAK2/STAT4/STAT5 signaling pathway by PBMT to regulate the function of peripheral CD4^+^ T lymphocytes has a certain contribute to promoting AHN, alleviating the pathological symptoms of AD, and ultimately rescuing cognitive impairment in AD mouse models, suggesting that PBMT has potential immunotherapeutic value in AD, which is likely reflected by regulating CD4^+^ T lymphocyte function.

## Materials and methods

### Mice

APP/PS1 transgenic mice (purchased from the Jackson Laboratory, expressing a chimeric mouse/human amyloid precursor protein bearing the Swedish mutation (Mo/HuAPP695swe) and a mutant human Presenilin 1 protein (PS1-dE9) in central nervous system neurons), 3xTg-AD transgenic mice (purchased from the Jackson Laboratory, homozygous for Tg (APPSwe, tauP301L) 1Lfa and homozygous for Psen1 < tm1Mpm >) and C57BL/6J mice (purchased from Guangdong Medical Laboratory Animal Center, Guangzhou, China), as wild-type (WT) mice, were used in this study. In detail, we used 6-month-old male mice for behavioral experiments and physiological index detection, 3- to 4-week-old WT and transgenic mice for splenic lymphocyte extraction, and 14-day embryonic (E14) WT and transgenic fetal mice for isolation and extraction of hippocampal neural stem cells. All of the mice were housed in the animal facilities of the Institute of Biophotonics, South China Normal University. All animal procedures and breeding are approved by the guidelines of The Care and Use of Laboratory Animals (Institute of Laboratory Animal Resources, Commission on Life Sciences, National Research Council). All animal experimental procedures are in accordance with rules dictated by the animal ethics Committee.

### Experimental design

In animal experiments to detect physiological index, we first randomly selected three groups of 6-month-old WT, APP/PS1 and 3xTg transgenic AD mice, with 12–16 mice in each group, the above three groups of mice were then randomly divided into six groups (WT, WT + PBMT, APP/PS1, APP/PS1 + PBMT, 3xTg, 3xTg + PBMT) with 6–8 mice in each group. The lymph nodes in WT + PBMT, APP/PS1 + PBMT and 3xTg + PBMT groups of mice were treated with PBMT (10 J/cm^2^, 10 min per day) for one month, considering the use of different experimental methods to detect the same physiological index, each group of mice was randomly divided into several subgroups for different experiments.

### Morris water maze (MWM) test

In the last week of PBMT treatment of mouse lymph nodes, the MWM was used to evaluate hippocampal-dependent spatial learning and memory as previously described [45, 46]. To summarize, the device was composed of a circular metal pool (100 cm in diameter) filled with opaque water at 22 ± 0.5 °C. The maze was divided into four quadrants, one of which had a transparent escape platform with a diameter of 10 cm, located 1.0 cm underwater. The circular maze was well lit, and there were obvious clues on the walls. The experimental training phase was carried out continuously for 5 days, training 4 times a day. During training, put the mice into the pool from four water entry points facing the pool wall, and the time required for the mice from entering the water to find the underwater hidden platform and stand on it is record as the incubation period, expressed in seconds (s). After the mice find the platform, let it stand on the platform for 10 s. If the mouse fails to find the platform within 60 s after entering the water, gently drag it from the water onto the platform and let it stay for 10 s. Each mouse was trained once from entering the water to finding the platform, and the time interval between each training was 15–20 min. After the training, the platform was removed on the 6th day, the mice were put into the water from a position diagonally away from the original platform, and mice were allowed to swim for 60 s to determine their search bias.

### PBMT treatments

PBMT treatment is performed as described in our previous work [34]. Cells were irradiated with a semiconductor laser (635 nm, NLFBA-2.0-635, nLight Photonics Corporation, Vancouver, WA, USA) under dark conditions for 5 min at a corresponding energy density of 2 J/cm^2^. In each experiment, the surrounding environment was kept completely dark or very dim to minimize any environmental interference. In animal experiments, 6-month-old WT, APP/PS1, and 3xTg-AD mice were treated with PBMT for 10 min daily for 1 month. This study calculated that the penetration rate through the superficial axillary adipose tissue to the axillary lymph nodes was about 20%, and the total laser energy density of 635 nm PBMT delivered to the axillary epidermis was 10 J/cm^2^, correspondingly, the energy density that passed through the superficial axillary adipose tissue to reach the lymph nodes was 2 J/cm^2^. The mouse axillary epidermis did not have any local temperature rise during irradiation. Please refer to Table S1-S2 for detailed information on laser parameters and treatment parameters used in in vivo and in vitro PBMT treatments.

### Splenic T lymphocytes extraction

T lymphocytes were harvested from mouse spleens and resuspended in ACK lysis buffer (0.15 M NH_4_Cl, 1.0 mM KHCO_3_, 0.1 mM Na_2_EDTA, pH 7.2–7.4) to remove red blood cells. Used the mouse spleen lymphocyte separation kit (LTS1092PK, TBDsciences, Tianjin, China) to extract and purify the cells [1], and all procedures were carried out in accordance with the instructions of the kit. The extracted primary T lymphocytes were resuspended in RPMI1640 containing 10% heat-inactivated fetal bovine serum (FBS), inoculated in a six-well plate and mixed uniformly, and then put the plate in a humidified incubator containing 5% CO_2_ at 37 ℃ for culture.

### Neural stem cell isolation

The NSCs used in this study were isolated from the hippocampus of C57BL/6J, APP/PS1 transgenic mice, and 3xTg-AD transgenic mice at E14. The specific isolation methods had been described in detail in our previous studies [34]. Briefly, we harvested the pups at E14, transferred the pups to a 10-cm sterile petri dish containing HBSS, then used tweezers to separate the pup’s head, discarded the skull, and then transferred each tissue culture dish to the dissecting microscope, dissected out the hippocampus to be used for establishing the culture, and transferred each harvested brain area to a 1.5-mL centrifuge tube containing 1-mL ice-cold HBSS. After washing the obtained tissue twice with HBSS, 0.05% trypsin was added to the tissue, and the tissue digested at 37 °C for 10 min and pipette several times, then digested for another 10 min, finally DMEM/F-12 containing 10% FBS was added to stop the digestion, the waste tissue was discarded after pipetting several times, the cell-containing medium was then centrifuged at 1800 rpm for 8 min, and precipitation is NSCs. NSCs were maintained in DMEM/F-12 medium containing 20 ng/mL basic fibroblast growth factor (bFGF), 20 ng/mL epidermal growth factor (EGF) and 2% B27. After culturing in a 5% CO_2_, 37 °C incubator for 3–4 days, neurospheres appeared. At this time, StemPro accutase was used to digest the neurospheres, and then seeded the digested NSC in a plate pre-coated with 0.6% Matrigel, the cells were cultured in a 5% CO_2_, 37 °C incubator, and half of the culture medium was replaced every two days.

### Western blotting

Tissues and cells were lysed on ice with lysis buffer (50 mM Tris–HCl, pH 8.0, 150 mM NaCl, 1% Triton X-100) containing complete protease inhibitor cocktail (5892791001, Roche) for 60 min, and then centrifugation at 12,000 rpm at 4 ℃ for 15 min. The supernatant was taken and the equal amounts of protein were electrophoretized with sodium-dodecyl sulfate polyacrylamide gel (SDS-PAGE) and blotted with the indicated antibodies. The antibodies used and their dilutions were as follows: anti-TGFβ1 (Novus, NBP2-22114, 1:1000); anti-IGF-1 (Novus, NBP2-34249, 1:500); anti-BDNF (Proteintech, 66292-1-lg, 1:1000); anti-β-Actin (Santa Cruz, sc-47778, 1:1000); anti-CD4 (RD, MAB554-SP, 1:1000); anti-p-JAK2 (CST, 4406, 1:1000); anti-p-STAT4 (Absin, abs131066, 1:1000); anti-p-STAT5 (CST, 4322, 1:1000); anti-T-bet (CST, 13232,1:1000); anti-Foxp3 (Abcam, ab215206, 1:1000); anti-Tuj1 (Proteintech, 66375-1-lg, 1:1000); anti-PSD95 (Proteintech, 20665-1-AP, 1:1000); Goat anti-Mouse IgG H&L (Alexa Fluor^®^ 680) (Abcam, ab175775, 1:10,000); Goat anti-Rabbit IgG H&L (Alexa Fluor^®^ 790) (Abcam, ab175781, 1:15,000). The detailed experimental procedures had been described in the previous work of our team [37]. The results of western blotting were analyzed with Odyssey and ImageJ software.

### Tissue preparation, immunohistochemistry and immunocytochemistry

The mice were euthanized by intraperitoneal injection of avertin, and then the mice were transcardially perfused with normal saline, harvested the brain tissue, subsequently, the brain tissue was fixed in 4% paraformaldehyde (PFA) overnight at 4 °C, and then used in sequence 15% and 30% sucrose solution gradient dehydration. The brains were embedded in Optimal Cutting Temperature (OCT) compound, and sliced into 10-μm-thick slices using a freezing microtome (Leica, CM1850). The tissue sections were sequentially fixed with 4% PFA, permeabilized with 0.5% Triton X-100 (surface antigen detection did not require this step), and blocked with 5% bovine serum albumin (BSA). Then, the sections were incubated overnight at 4 °C in appropriately diluted primary antibodies containing PBS with 0.5% Triton X-100, and then Alexa Fluor 488/555/647 conjugated secondary antibody was used to detect the primary antibody. The experimental method of immunocytochemistry was similar to that of immunohistochemistry, after PBMT, the cells were fixed with 4% PFA for 15 min, permeabilized with 0.2% Triton X-100 for 30 min, and blocked with 5% BSA for 1 h at room temperature. After blocking, the cells were incubated with the primary antibody overnight at 4 °C, and then incubated with the corresponding fluorescent secondary antibody at room temperature for 2 h. Finally, the nuclei were stained with DAPI. The primary, secondary antibodies, and their dilutions were as follows: anti-Nestin (Proteintech, 19483-1-AP, 1:100), anti-Tuj1 (Proteintech, 66375-1-lg, 1:300); anti-GFAP (CST, 3670, 1:300); anti-Iba-1 (CST, 17198, 1:300); anti-Aβ (Biolegend, 109902, 1:300); anti-CD4 (RD, MAB554-SP, 1:50); anti-PSD95 (Proteintech, 20665-1-AP, 1:300); anti-Lamp1 (Abcam, ab208943, 1:100); Goat anti-Mouse IgG H&L (Alexa Fluor^®^ 488) (Abcam, ab150113, 1:400); Goat anti-Rabbit IgG H&L (Alexa Fluor^®^ 555) (Abcam, ab150078, 1:400); Goat anti-Rat IgG H&L (Alexa Fluor^®^ 647) (Abcam, ab150159, 1:400), and DAPI for cells nuclear staining (Sigma, D9542-1MG). After staining, the sections were coverslipped and kept at 4 °C in the dark until further analysis. Laser scanning confocal microscope (Zeiss, LSM880) was used to obtain immunofluorescence images of each treatment group. ZEN blue and ImageJ software were used to analyze the images.

### Flow cytometry

For flow cytometric assessment of the functional property of T lymphocytes in vitro, cells were fixed with 4% PFA and permeabilized with 0.2% Triton X-100 after surface marker staining. For flow cytometric assessment of tissue cells in vivo, the tissues to be studied were cut off after euthanizing the mice, the tissues were cut into pieces with scissors and digested with accutase. Then, the tissues were digested into single cells, which was terminated with PBS, and tissue cells were obtained by centrifugation. The cells were stained with fluorescent-labeled antibodies targeting cell surface markers at room temperature for 1 h, then fixed with 4% PFA, permeabilized with 0.2% Triton X-100, and incubated with fluorescent-labeled antibodies targeting intracellular antigens at room temperature for 1 h. After incubation, the cells were cleaned with PBS and filtered for flow cytometry detection. If the primary antibody was not labeled with fluorescence, fluorescent secondary antibody was used to identify the primary antibody. The specific procedure was according to the manufacturer's instructions. The antibodies used for flow cytometry were as follows: BB700 anti-mouse CD4 (BD, 566408); APC anti-mouse IFN-γ (BD, 562018); FITC anti-mouse CD69 (BD, 561929); PE anti-mouse IL-10 (BD, 561060); anti-Tuj1 (Proteintech, 66375-1-lg); anti-Nestin (Proteintech, 19483-1-AP); anti-AMPAR (Proteintech, 11994-1-AP); anti-PSD95 (Proteintech, 20665-1-AP); anti-Iba-1 (CST, 17198); anti-TGFβ1 (Novus, NBP2-22114); anti-BDNF (Proteintech, 66292-1-lg); anti-IGF-1 (Novus, NBP2-34249). Staining cell data were obtained using a flow cytometer (Beckman, A00-1-1102) and analyzed using CytExpert and FCS Express software.

### Enzyme-linked immunosorbent assay (ELISA)

Using IFN-γ ELISA kit (Mlbio, ml002277-2) and IL-10 ELISA kit (Mlbio, ml002285-2) to measure the concentration of IFN-γ and IL-10, respectively, in the brain and serum of mouse and T cell supernatants, using BDNF ELISA kit (Mlbio, ml002219-2), IGF-1 ELISA kit (Mlbio, ml002288-2) and TGFβ1 ELISA kit (Mlbio, ml002115-2) to measure the concentration of BDNF, IGF-1 and TGFβ1, respectively, in brain of mouse. All procedures of the above assay were performed according to the manufacturer's manuals.

### Detection of reactive oxygen species (ROS)

T lymphocytes were divided into different experimental groups. NAC (Sigma, A9165, 1 mM) was added to the cell culture medium at 1 h before PBMT treatment. After 0.5 h, DCF probe (Beyotime, S0033, 40 μM) was added. After PBMT treatment 30 min, the cells were collected and stained with CD4 antibody, and then washed with PBS. Finally, staining cells were detected using flow cytometer and analyzed using CytExpert and FCS Express software.

### Statistical analysis

GraphPad (prism 8.0) and Origin 6.0 were used for statistical analyses, Two-way analysis of variance (ANOVA) was used for escape latency analysis. The other comparisons between two or among more groups were determined by one-way ANOVA and two-tailed unpaired Student’s *t* tests. All data were presented as the mean ± standard error of the mean (SEM), and *P* values < 0.05 was considered statistically significant (see each figure for details). The sample sizes are indicated in the figure legends.

## Results

### PBMT-treated lymph nodes attenuated cognitive deficits in APP/PS1 and 3xTg-AD mice

**Fig. 1:**
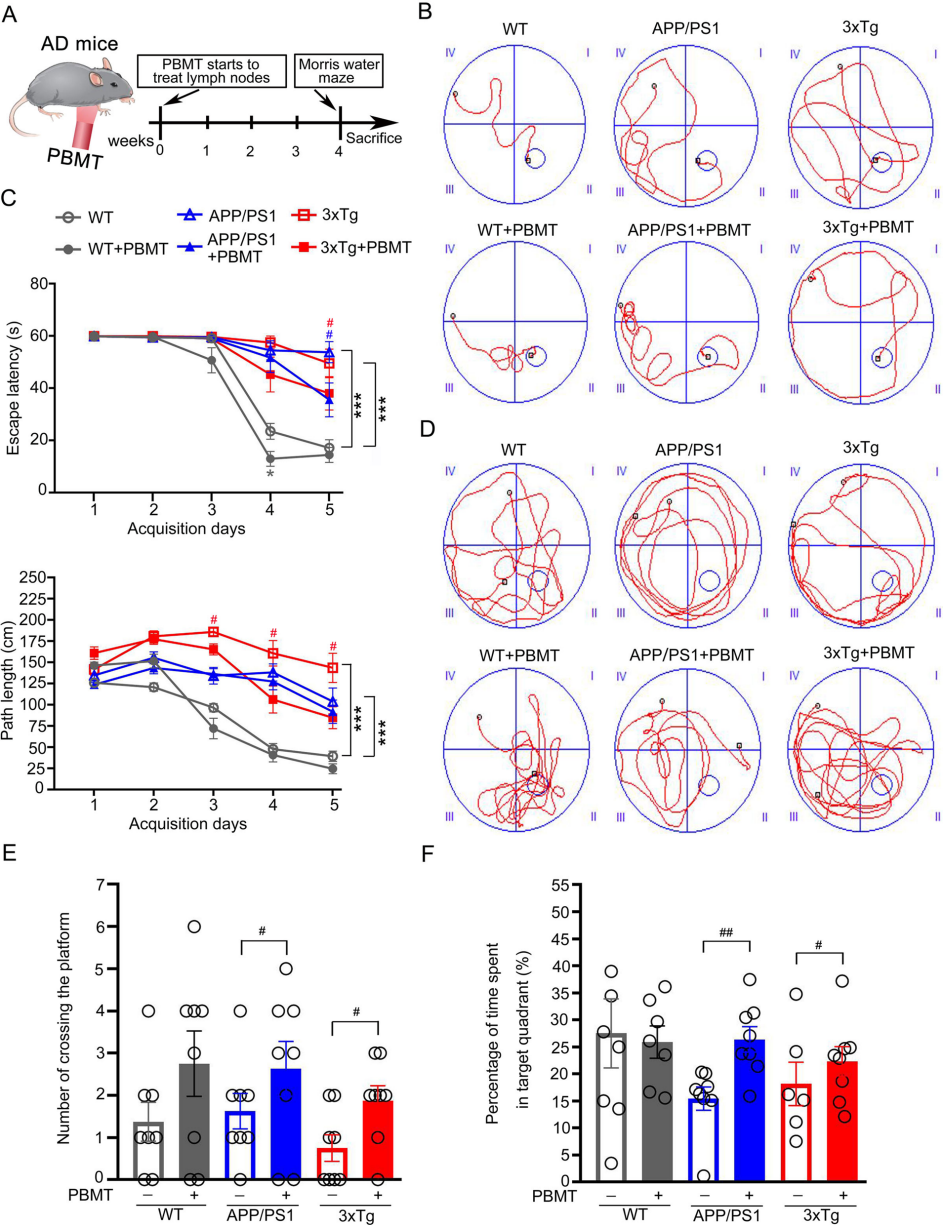
The treatment of lymph nodes by PBMT attenuated cognitive deficits in APP/PS1 and 3xTg-AD mice. **A** The experimental procedure. **B** Representative swimming paths on day 5 during the acquisition phase. **C** The daily escape latency and the length of the path to the platform were recorded during training. **D** Representative swimming traces of six groups mice after the WMW test. **E**, **F** The number of crossing the platform of each group mice (**E**) and the percentage of time spent in target quadrant (**F**) of the original platform location during the 60 s of the MWM test. All data were detected by the MWM test. All quantifications are presented as mean ± SEM and were analyzed by one-way ANOVA test; ***p < 0.001 versus WT group, #*p* < 0.05, ##*p* < 0.01 versus indicated group (*n* = 6–8 mice per group)

To detect whether PBMT treatment of lymph nodes affects spatial learning/memory in APP/PS1 and 3xTg-AD mice, we performed the MWM task [47]. The experiment was conducted according to experimental procedure (Fig. 1A), spatial learning ability of mice was evaluated by the time it took to find the hidden platform (escape latency) during the 5-day acquisition training. During the acquisition phase, representative swimming paths on day 5 showed significant differences among groups (Fig. 1B). During training, mice in all groups showed a daily decline in escape latency, and the decline was more significant with increasing training days (Fig. 1C). Moreover, the path length for APP/PS1 and 3xTg-AD mice to find the platform was longer than that of PBMT-treated mice, respectively (Fig. 1C), suggesting that PBMT-treated lymph nodes of AD mice significantly improved learning deficits. In the probe trial phase, PBMT-treated AD mice performed better than non-PBMT-treated AD mice (Fig. 1D). Meanwhile, compared with the respective untreated APP/PS1 and untreated 3xTg-AD mice, the PBMT-treated mice crossed over the former platform location more frequently (Fig. 1E) and spent longer time in the target quadrant (Fig. 1F). Together, these data showed that PBMT-treated lymph nodes improved the spatial learning and memory ability in AD mice.

### PBMT upregulated TGFβ1/IGF-1/BDNF expression and promoted AHN in the brains of AD mice

**Fig. 2:**
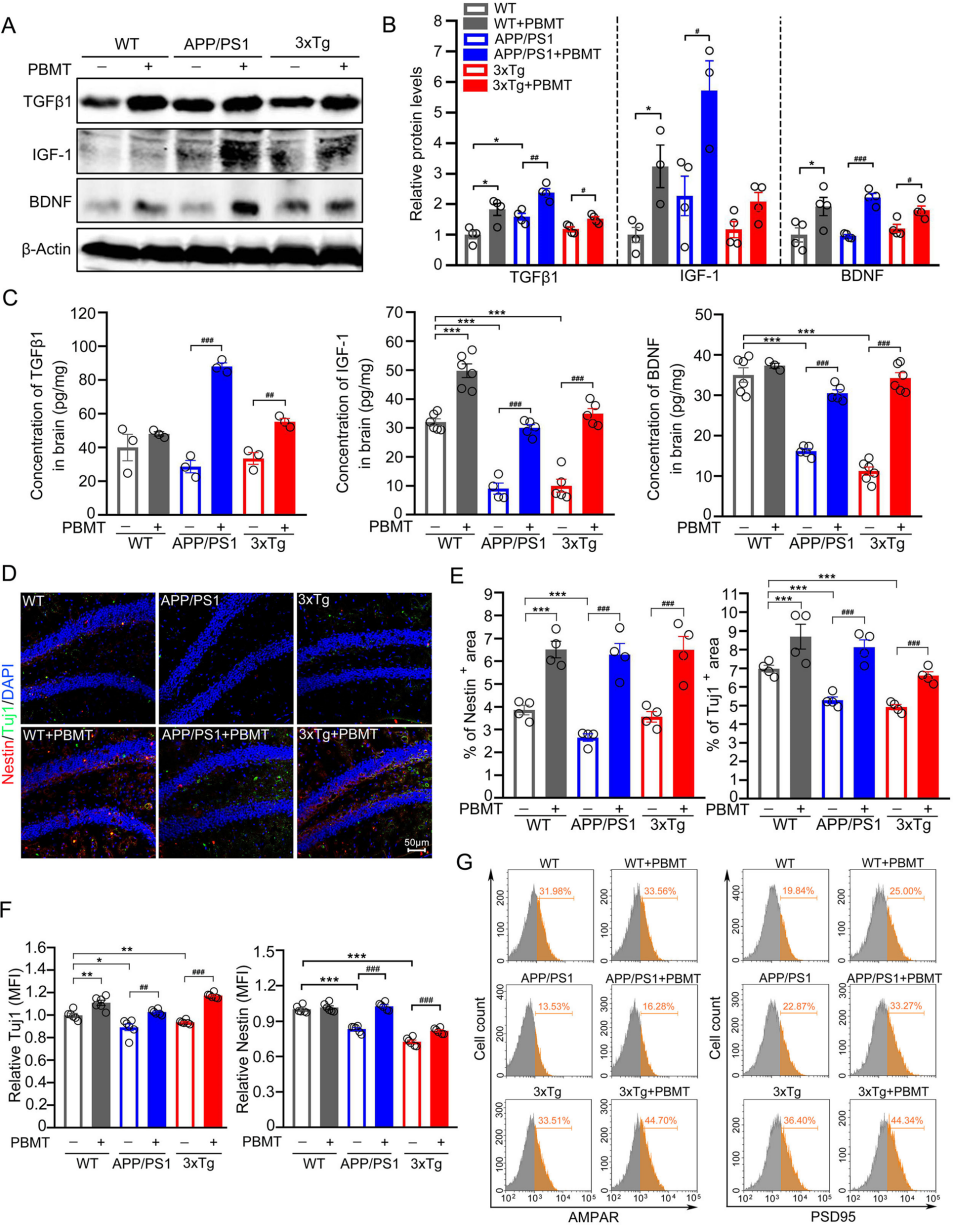
The upregulation of TGFβ1/IGF-1/BDNF expression in the brain of AD mice by PBMT extended to promote adult hippocampal neurogenesis. **A**, **B** Western blotting analysis (**A**) and quantification (**B**) of TGFβ1/IGF-1/BDNF expression in APP/PS1 and 3xTg-AD mouse brain after PBMT, (*n* = 3–4 per group). **C** The concentration of TGFβ1/IGF-1/BDNF in brain tissues were measured by enzyme linked immunosorbent assay (ELISA), (*n* = 3–6 per group). **D** Representative images of Nestin^+^ (neural stem cell staining) and neuronal class-III β-tubulin (Tuj1)^+^ (newborn neurons staining) expression cells in APP/PS1 and 3xTg-AD mouse hippocampal dentate gyrus (DG) at the end of PBMT. Scale bars, 50 μm. **E** Quantitative analyses of Nestin^+^ and Tuj1^+^ area in the hippocampal DG of each group, (*n* = 4 per group). **F** Quantitative analyses of the Nestin and Tuj1 mean fluorescence (MFI) in the brain tissues of each group after PBMT. The Nestin and Tuj1 MFI were detected by flow cytometer, (*n* = 5–6 per group). **G** Tuj1 antibody was used to staining the newborn neurons, and then, the expression of α-amino-3-hydroxy-5-methyl-4-isoxazole-propionic acid receptors (AMPAR) and postsynaptic density protein 95 (PSD95) on Tuj1^+^ neurons were detected by flow cytometer. All quantifications are presented as mean ± SEM and were analyzed by one-way ANOVA test; ***p < 0.001, **p < 0.01, *p < 0.05 versus WT group; ###*p* < 0.001, ##*p* < 0.01, #*p* < 0.05 versus indicated group

At the end of WMW test, mice were killed and brain tissues were harvested to detect physiological indicators. The expression of TGFβ1/IGF-1/BDNF in the brains of AD mice were detected via different experimental methods, we observed that these three proteins were significantly upregulated in PBMT-treated lymph nodes versus untreated APP/PS1 mice. We also detected TGFβ1/IGF-1/BDNF levels in another AD mouse model, 3xTg-AD mice, and found similar results to APP/PS1 mice after PBMT-treated lymph nodes (Fig. 2A–C; Additional file 1: Fig. S1A–C). To further explore whether changes in the content of these microenvironmental components in the brain affect AHN, immunostaining with NSCs specific antibody Nestin and immature neurons specific antibody Tuj1 (Fig. 2D) revealed a significantly increase in cells number in hippocampal subgranular zone (SGZ) of both APP/PS1 and 3xTg-AD mice after PBMT (Fig. 2E). Similarly, the protein expression levels of Nestin and Tuj1 were also upregulated after PBMT treatment in the brain tissues of these two AD models (Fig. 2F). To examine the function of newborn neurons, we saw increased cell numbers of α-amino-3-hydroxy-5-methyl-4-isoxazole-propionic acid receptors (AMPARs)^+^ on Tuj1^+^ cells (APP/PS1 + PBMT: 16.28%; APP/PS1: 13.53%; 3xTg-AD + PBMT: 44.70%; 3xTg-AD: 33.51%) and postsynaptic density protein 95 (PSD95) ^+^ on Tuj1^+^ cells (APP/PS1 + PBMT: 33.27%; APP/PS1: 22.87%; 3xTg-AD + PBMT: 44.34%; 3xTg-AD: 36.40%) after PBMT-treated lymph nodes of AD mice (Fig. 2G). Collectively, these results indicated that lymph nodes of multiple AD mouse models treated with PBMT improved the brain microenvironment to promote AHN, and the typical proteins associated with neuronal function were upregulated in the newborn neurons.

### PBMT alleviated neuroinflammation, reduced reactive astrogliosis, and increased the concentration of IFN-γ/IL-10 in APP/PS1 and 3xTg-AD mice

**Fig. 3:**
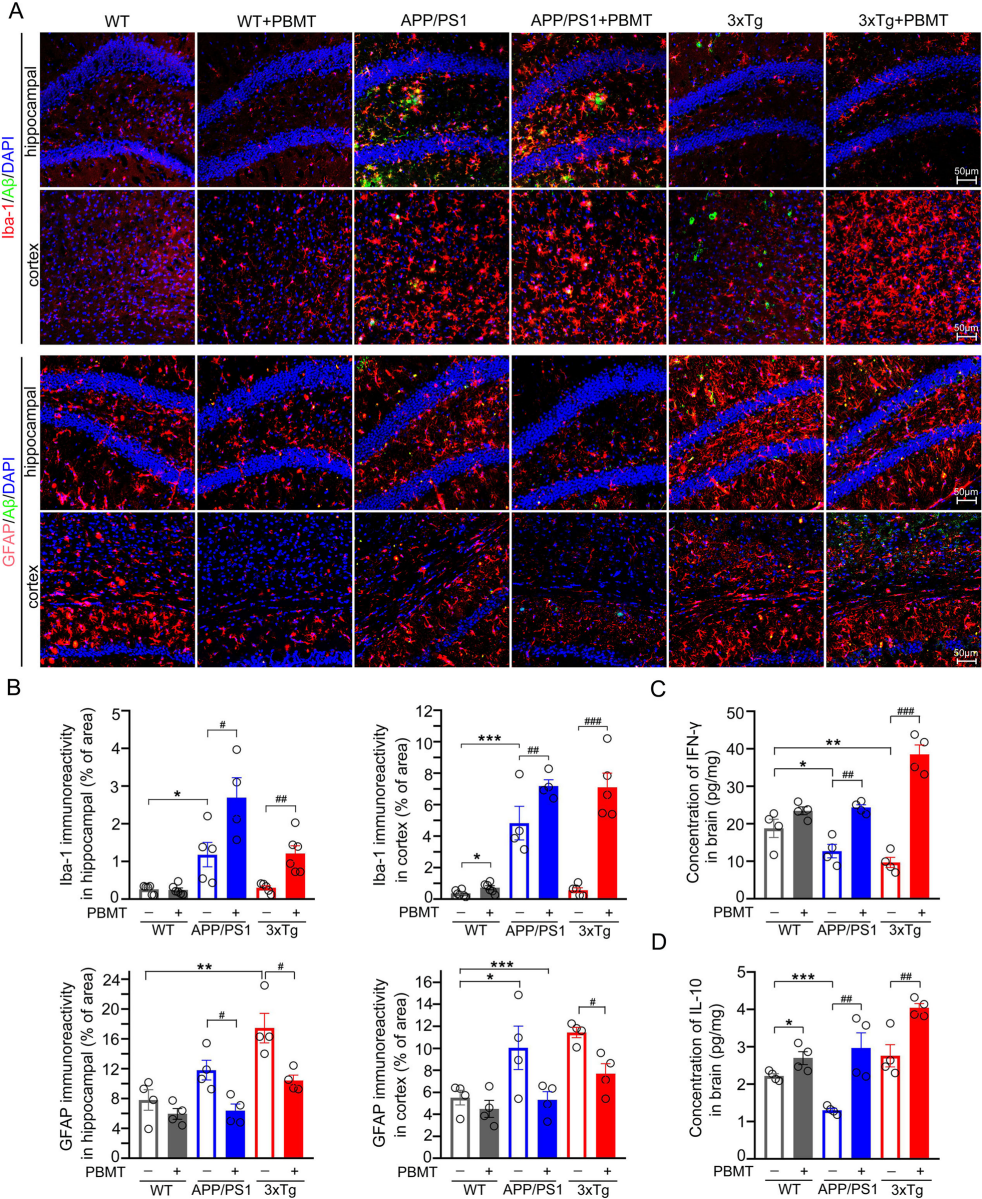
PBMT-treated lymph nodes affected neuroinflammation, reactive astrogliosis, and concentration of IFN-γ/IL-10 in the brain tissue of APP/PS1 and 3xTg-AD mice. **A**, **B** Representative images (**A**) and quantitative analyses (**B**) of Iba-1^+^ (activated microglia staining), GFAP^+^ (reactive astrocyte staining), and Aβ (Aβ plaque staining) in cortex and hippocampal regions from PBMT-treated or un-treated APP/PS1 and 3xTg-AD groups. DAPI was used to counterstain nuclei. Scale bar: 50 μm, (*n* = 4–6 per group). **C**, **D** ELISA was used to detect the concentration of IFN-γ (**C**) and IL-10 (**D**) in the brain tissue of APP/PS1 and 3xTg-AD groups after PBMT-treated lymph nodes, (*n* = 4 per group). All quantifications are presented as mean ± SEM and were analyzed by One-way ANOVA test; ***p < 0.001, **p < 0.01, *p < 0.05 versus WT group; ###*p* < 0.001, ##*p* < 0.01, #*p* < 0.05 versus indicated group

Activated microglia could not only phagocytose fibrous beta-amyloid (fAβ) or dead cells from the CNS, but also secrete different neurotrophic factors to promote neuronal survival [48]. In the neuroinflammation process of AD pathology, reactive astrocytes could promote the death of neurons by controlling the uptake and release of neurotransmitters [49], resulting in the disorder of the entire brain microenvironment and thereby inhibiting neurogenesis. Therefore, we further explored whether PBMT-treated lymph nodes of AD mice affected neuroinflammation and reactive astrogliosis in the brain. Surprisingly, data showed that the number of ionized calcium binding adaptor molecule-1 (Iba-1) ^+^ cells in brain tissue was significantly increased after PBMT-treated lymph nodes (APP/PS1 + PBMT: 5.17%; APP/PS1: 2.99%; 3xTg + PBMT: 5.08%; 3xTg: 1.87%) (Additional file 1: Fig. S1D, E), and then, we immunostained Aβ and Iba-1^+^ cells in hippocampal DG and cortex. The results showed that compared with untreated controls, the number of microglia and cell body was increased in these two regions, and additionally, the deposition of Aβ was decreased (Fig. 3A, B). Furthermore, immunostaining with astrocyte-specific antibody glial fibrillary acidic protein (GFAP) (Fig. 3A) and dystrophic neurites-specific antibody recombinant lysosomal associated membrane protein 1 (lamp1) (Additional file 1: Fig. S1F) revealed significantly decreased reactive astrogliosis (Fig. 3B) and dystrophic neurites (Additional file 1: Fig. S1G) in the brain of APP/PS1 mice after PBMT, versus untreated controls. We also examined the above physiological indicators in another AD model mouse after PBMT treatment, 3xTg-AD mice, the results were consistent with our findings in APP/PS1 mice (Fig. 3A, B; Additional file 1: Fig. S1F, G). Subsequently, the concentration of IFN-γ/IL-10 in the brain tissue and serum of APP/PS1 mice was assessed by ELISA, we observed PBMT significant changed IFN-γ/IL-10 levels, versus untreated APP/PS1 mice (Fig. 3C, D; Additional file 1: Fig. S2A, B). Beyond APP/PS1 mouse model, we also performed the same analysis in 3xTg-AD mice. In line with our findings in APP/PS1 mice, we detected significantly increased IFN-γ/IL-10 levels in brain after PBMT-treated lymph nodes (Fig. 3C, D; Additional file 1: Fig. S2A, B). The above results indicated that the lymph nodes of APP/PS1 and 3xTg-AD mice treated with PBMT reduced neuroinflammation and reactive astrogliosis, which were likely to be related to the increase of the concentration of immune factors IFN-γ/IL-10 in brain tissue.

### PBMT upregulated the expression of IFN-γ/IL-10 in CD4^+^ T cells and enhanced the recruitment of CD4^+^ T cells to brain in two mouse models of AD

**Fig. 4:**
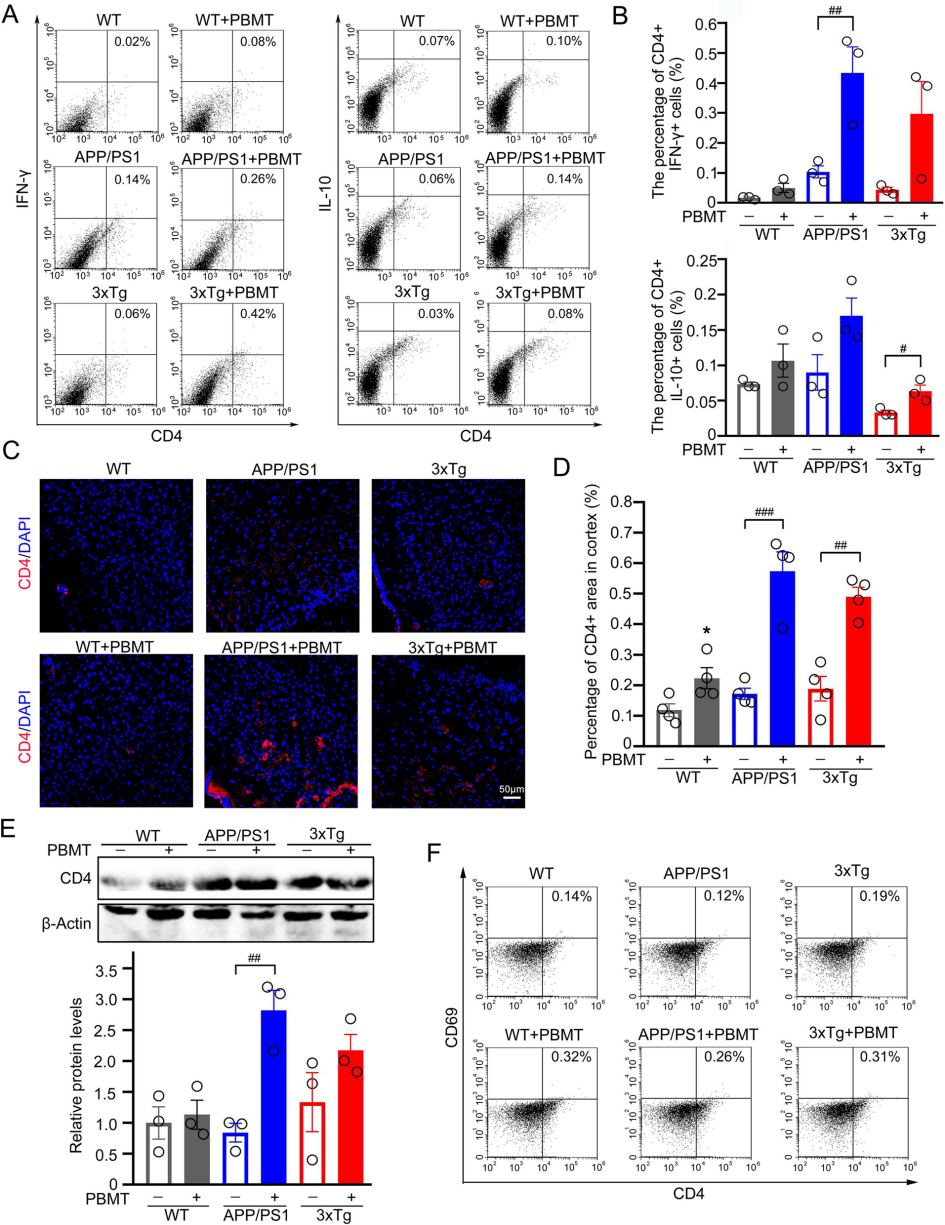
PBMT treatment of lymph nodes affected the activation of CD4^+^ T cells, the expression of IFN-γ/IL-10 in CD4^+^ T cells, and the recruitment of CD4^+^ T cells to the brain of APP/PS1 and 3xTg-AD mice. **A**, **B** Flow cytometry was used to detect the number of CD4^+^ IFN-γ^+^ T cells and CD4^+^ IL-10^+^ T cells in APP/PS1 and 3xTg-AD mouse brain tissues after PBMT treatment (*n* = 3 per group) (**A**) and its quantitative analyses (**B**). **C**, **D** Representative images (**C**) and quantitative analyses (**D**) of CD4^+^ T cells in cortex regions from PBMT-treated or un-treated APP/PS1 and 3xTg-AD groups. Nuclei was stained by DAPI. Scale bar: 50 μm, (*n* = 4 per group). **E** Western blotting analysis and quantification of CD4 protein expression in APP/PS1 and 3xTg-AD mouse brains with or without PBMT, (*n* = 3 per group). **F** The number of CD4^+^ CD69^+^ T cells in the brain tissue of six groups was detected by flow cytometry. The statistical analysis of **F** was provided in Additional file 1: Fig. S3A. All quantifications are presented as mean ± SEM and were analyzed by One-way ANOVA test; *p < 0.05 versus WT group; ###*p* < 0.001, ##*p* < 0.01, #*p* < 0.05 versus indicated group

Subsequently, in order to explore whether the increase of IFN-γ/IL-10 concentration in serum and brain tissue caused by PBMT treatment of lymph nodes from APP/PS1 and 3xTg-AD mice were related to CD4^+^ T cells, we performed flow cytometry to detect the number of CD4^+^ IFN-γ^+^ T cells and CD4^+^ IL-10^+^ T cells in brain and spleen of each group. The results showed PBMT increased the number of CD4^+^ IFN-γ^+^ T cells in the brain tissue (APP/PS1 + PBMT: 0.26%; APP/PS1: 0.14%; 3xTg-AD + PBMT: 0.42%; 3xTg-AD: 0.06%), versus no treatment controls (Fig. 4A, B), and the number of CD4^+^ IL-10^+^ T cells in the brain tissue were also increased by PBMT (APP/PS1 + PBMT: 0.14%; APP/PS1: 0.06%; 3xTg-AD + PBMT: 0.08%; 3xTg-AD: 0.03%), compared with untreated groups (Fig. 4A, B). Spleen tissue was used to carry out similar analysis after PBMT-treated lymph nodes of AD transgenic mice; we saw the consistent results with our findings in brain (Additional file 1: Fig. S2C–F). Meanwhile, we analyzed CD4 immunostaining in the brain tissue of APP/PS1 mice after 4 weeks of PBMT treatment (Fig. 4C). We observed an extremely significant increase in CD4^+^ T cells, versus untreated controls (Fig. 4D). We also extended the mouse model to 3xTg-AD mice, the results of CD4^+^ T cells immunofluorescence in line with our finding in APP/PS1 mice, but the difference was not as significant as APP/PS1 mouse group after PBMT treatment (Fig. 4C, D). The data of western blotting for CD4 protein in brain tissues of these two mouse models were consistent with CD4 immunostaining (Fig. 4E). Moreover, we also detected a significant increase in the number of CD4^+^ CD69^+^ T cells in the brain tissue of APP/PS1 mice (APP/PS1 + PBMT: 0.26%; APP/PS1: 0.12%) and 3xTg-AD mice (3xTg-AD + PBMT: 0.31%; 3xTg-AD: 0.19%) after PBMT treatment (Fig. 4F; Additional file 1: Fig. S3A). These results indicated that PBMT-treated lymph nodes of AD mice promoted the activation of CD4^+^ T cells and enhanced the recruitment of CD4^+^ T cells to the brain.

### The activation of JAK2/STAT4/STAT5 by PBMT-induced ROS generation in CD4^+^ T cells was necessary for PBMT to up-regulate IFN-γ/IL-10

**Fig. 5:**
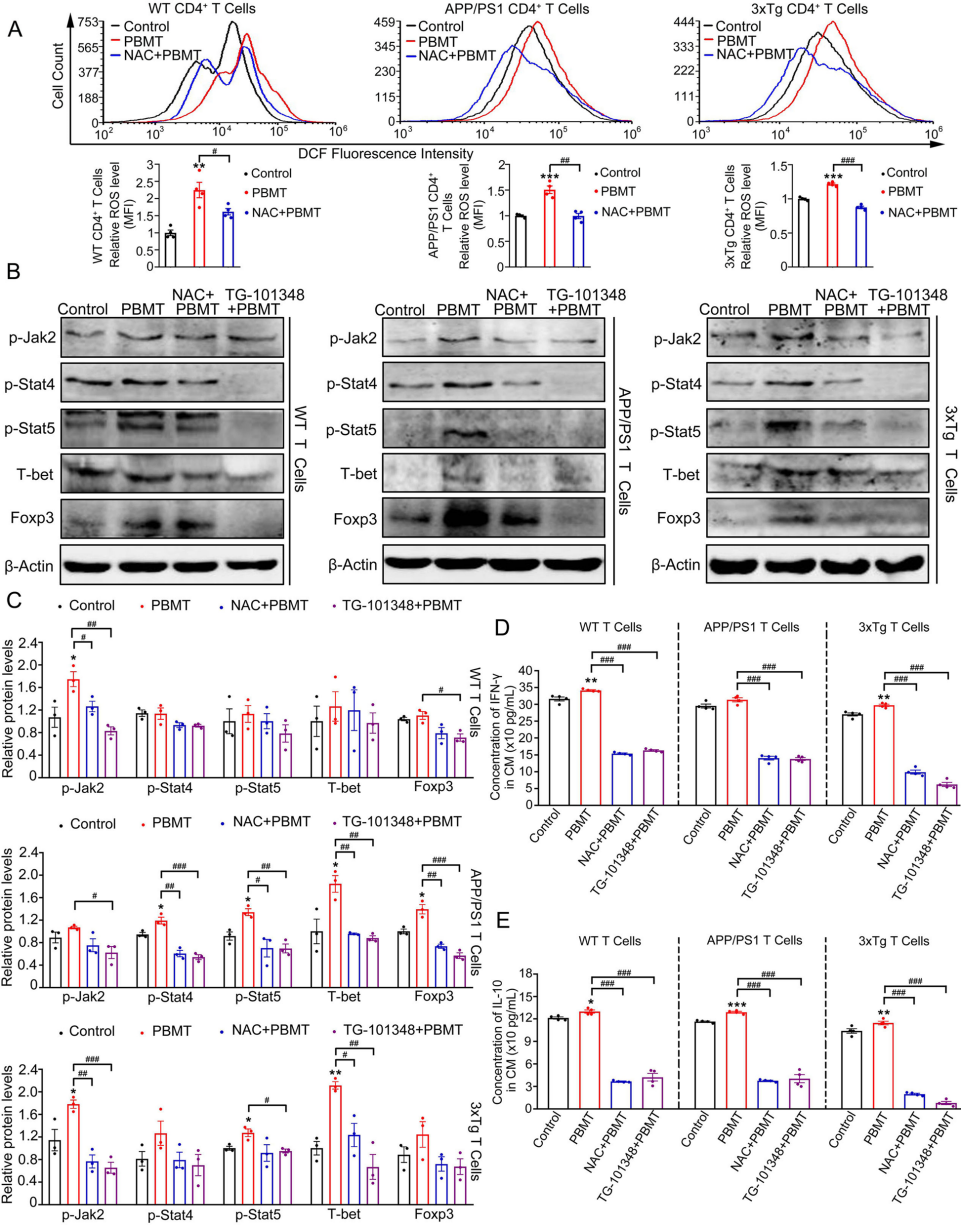
PBMT-induced ROS generation in CD4^+^ T cells activated JAK2/STAT4/STAT5 signal pathway to upregulate the expression of IFN-γ/IL-10. **A** CD4 antibody was used to staining the CD4^+^ T cells, and then the flow cytometry was used to detect and quantitative analyze the generation of ROS (revealed by DCF) in WT, APP/PS1, and 3xTg-AD CD4^+^ T cells with or without PBMT (*n* = 4 per group). Cells were pre-incubated with N-acetyl cysteine (NAC, 1 mM) before PBMT. **B**, **C** Western blotting analysis (**B**) and quantification (**C**) of the expression of p-JAK2, p-STAT4, p-STAT5, T-bet, Foxp3 in T cells from WT, APP/PS1, and 3xTg-AD with or without PBMT (*n* = 3 per group). Some cells were pre-incubated with NAC or TG-101348 (JAK2 inhibitor, MCE, HY-10409, 1 μM) before PBMT. **D**, **E** The concentration of IFN-γ (**D**) and IL-10 (**E**) in T cells from WT, APP/PS1, and 3xTg-AD conditioned medium (CM) were detected by ELISA after PBMT (*n* = 4 per group). All quantifications are presented as mean ± SEM and were analyzed by One-way ANOVA test; ***p < 0.001, **p < 0.01, *p < 0.05 versus control group; ###*p* < 0.001, ##*p* < 0.01, #*p* < 0.05 versus indicated group

To investigate the possible mechanism of PBMT responsible for the upregulation of IFN-γ/IL-10 in CD4^+^ T cells, spleen T lymphocytes from WT, APP/PS1 and 3xTg-AD mice were extracted to study the mechanism, respectively. It was found that ROS production in CD4^+^ T cells from WT, APP/PS1 and 3xTg-AD mice was significantly increased after PBMT treatment (Fig. 5A). Immediately, we detected the protein expression levels of p-JAK2, IL-10 transcriptional factor Foxp3, and transcriptional co-activator p-STAT5 in each group of T lymphocytes. Subsequently, we also detected the protein expression levels of IFN-γ transcriptional factor T-bet and transcriptional co-activator p-STAT4 in each group of T lymphocytes. As expected, the protein expression levels of p-JAK2, p-STAT4, p-STAT5, T-bet, and Foxp3 were increased in PBMT-treated T lymphocytes from WT, APP/PS1, and 3xTg-AD, respectively. However, these effects disappeared after adding NAC or TG-101348 (JAK2 inhibitor) (Fig. 5B, C).

Furthermore, we used ELISA to detect the secretion of IFN-γ/IL-10 by T cells from WT, APP/PS1 and 3xTg-AD under the corresponding different treatment conditions, separately. Consistent with the phosphorylation of JAK2, STAT4, STAT5, the expression of T-bet and Foxp3, PBMT treatment of T lymphocytes promoted the secretion of IFN-γ/IL-10 cytokines in each group (Fig. 5D, E). Collectively, our data demonstrated that PBMT-induced ROS generation in CD4^+^ T cells activated the JAK2/STAT4/STAT5 signaling pathway to promote the expression of transcription factor T-bet/Foxp3, thereby upregulating the secretion of IFN-γ/IL-10.

### The co-culture of NSCs with PBMT-treated T lymphocyte conditioned medium promoted NSCs differentiation and PSD95 expression in newborn neurons in vitro

**Fig. 6:**
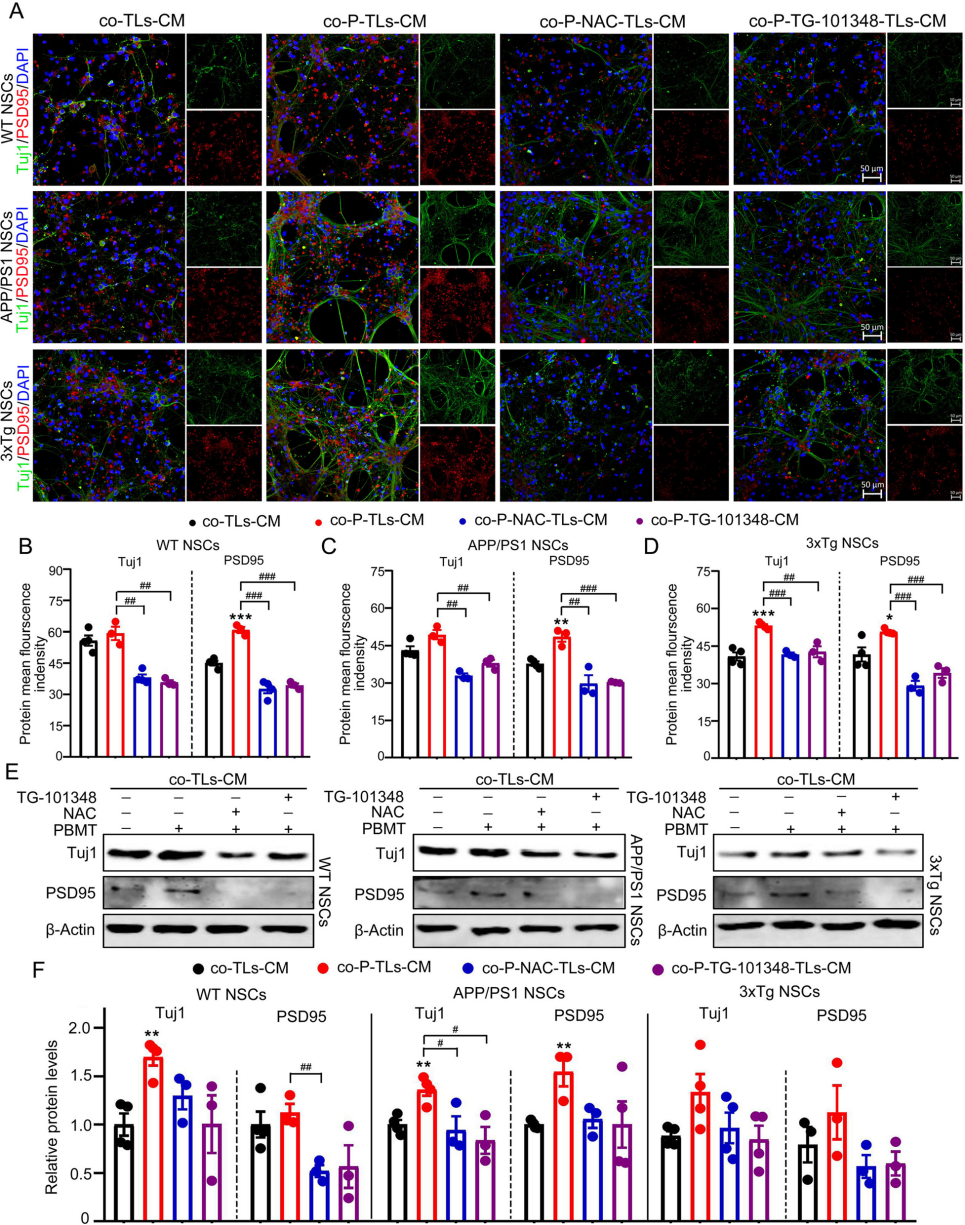
Co-culture of NSCs with PBMT-treated condition medium from T lymphocyte promoted the differentiation of NSCs and the expression of PSD95 in newborn neurons. **A** Representative images of Tuj1^+^ and PSD95^+^ expression cells in WT NSCs, APP/PS1 NSCs and 3xTg-AD NSCs after co-culturing with their respective T lymphocyte supernatants, DAPI was used to stain nuclei, Scale bars, 50 μm. **B**–**D** Quantitative analyses of Tuj1 and PSD95 protein MFI in WT NSCs (**B**), APP/PS1 NSCs (**C**), 3xTg-AD NSCs (**D**) after co-culturing with their respective T lymphocyte supernatants, the detection time and culture process were consistent with Fig. 6A (*n* = 3–4 per group). **E**, **F** Western blotting analysis (**E**) and quantification (**F**) of the expression of Tuj1 and PSD95 from WT NSCs, APP/PS1 NSCs, and 3xTg-AD NSCs after co-culturing with their respective T lymphocyte supernatants, the detection time and culture process were consistent with Fig. 6A (*n* = 3–4 per group). The NSCs of each group continued to differentiate until the 14th day before detecting, the ratio of T lymphocyte conditioned medium to NSCs differentiation medium was 1:4. The treatment groups of NSCs as follows: co-cultivate with T Lymphocytes conditioned medium (co-TLs-CM), co-cultivate with PBMT-treated T Lymphocytes conditioned medium (co-P-TLs-CM), co-cultivate with PBMT-treated NAC pre-incubated T Lymphocytes conditioned medium (co-P-NAC-TLs-CM), co-cultivate with PBMT-treated TG-101348 pre-incubated T Lymphocytes conditioned medium (co-P-TG-101348-TLs-CM). All quantifications are presented as mean ± SEM and were analyzed by one-way ANOVA test; ***p < 0.001, **p < 0.01, *p < 0.05 versus co-TLs-CM group; ###*p* < 0.001, ##*p* < 0.01, #*p* < 0.05 versus indicated group

In order to further explore whether IFN-γ/IL-10 secreted by CD4^+^ T lymphocytes actually affected AHN, we extracted hippocampal NSCs from WT, APP/PS1, and 3xTg-AD fetal mice (E14d), respectively. These extracted NSCs were co-cultured with conditioned medium (CM), containing WT, APP/PS1, and 3xTg-AD mouse T lymphocyte secretion factors, for NSC differentiation experiments. Splenic T lymphocytes extracted from WT, APP/PS1 and 3xTg-AD were set as: Control group, PBMT group, NAC + PBMT group, TG-101348 + PBMT group, then NSCs were co-cultured with T lymphocyte supernatants of different treatment groups. After co-cultured 14 days, immunocytochemical experiments (Fig. 6A–D) were performed and results showed that after WT NSCs, APP/PS1 NSCs and 3xTg-AD NSCs had co-cultured with their respective PBMT-treated T lymphocyte supernatants, the expression levels of Tuj1 and PSD95 were increased, however, after co-cultivating with PBMT-treated NAC or TG-101348 pre-incubated T Lymphocytes CM, the expression levels of Tuj1 and PSD95 were lower than PBMT-treated T Lymphocytes conditioned medium (co-P-TLs-CM) group, and the results of western blotting were consistent with immunocytochemical results (Fig. 6E, F). In addition, we also found an increase in the expression levels of Tuj1 and PSD95 in the brain tissues of APP/PS1, 3xTg-AD mice after PBMT-treated lymph nodes (Additional file 1: Fig. S3B, C). Taken together, these results indicated that PBMT treatment of CD4^+^ T lymphocytes promoted the secretion of IFN-γ/IL-10 cytokines, which had a certain regulatory effect on the differentiation of NSCs into neurons and the formation of synapses in newborn neurons to promote AHN in the AD mouse model.

## Discussion

**Fig. 7:**
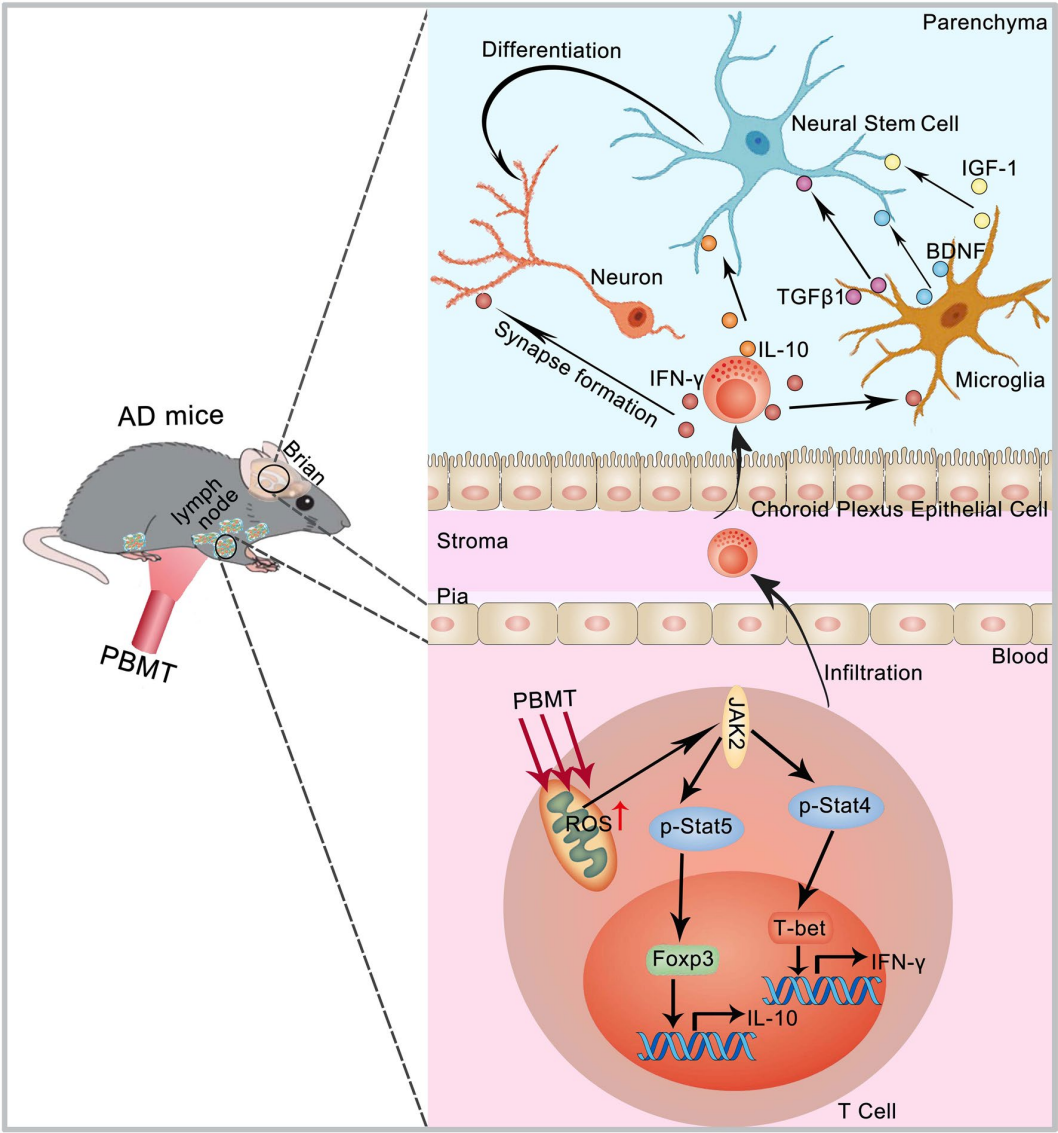
Schematic representation for promoted CD4^+^ T cell-derived IFN-γ/IL-10 by photobiomodulation therapy modulates neurogenesis to ameliorate cognitive deficits in APP/PS1 and 3xTg-AD mice

In this study, we found that PBMT treatment of APP/PS1 and 3xTg-AD mice lymph nodes promoted AHN to improve cognitive deficits, these effects mainly due to the upregulation of IFN-γ/IL-10 protein expression in brain tissue after PBMT treatment. Specifically, we demonstrated that PBMT treatment of lymph nodes in these two mouse models induced ROS production in CD4^+^ T cells, thereby activated the JAK2/STAT4/STAT5 signaling pathway, and then upregulated the expression of IFN-γ/IL-10. These CD4^+^ T cells with altered functions after PBMT treatment may be recruited to the brain tissue through blood circulation, and interact directly or indirectly with glial cells and NSCs. Subsequently, increased the content of TGFβ1/IGF-1/BDNF in the brain tissue, which improved the microenvironment of the NSCs niche to promote AHN and synaptic plasticity of newborn neurons (Fig. 7). More importantly, by co-cultivating the PBMT-treated conditioned medium of T lymphocytes from APP/PS1 and 3xTg-AD with the corresponding NSCs derived from APP/PS1 and 3xTg-AD, we found that the co-culture of PBMT-treated T lymphocyte conditioned medium with NSCs promoted the differentiation of NSCs and upregulated the expression of Tuj1 and PSD95. Thus, our results indicated that PBMT treatment of lymph nodes promoted the expression of IFN-γ/IL-10 in non-parenchymal CD4^+^ T cells by activating the JAK2/STAT4/STAT5 signaling pathway, and induced the improvement of brain microenvironmental conditions, thereby playing a beneficial role in neurogenesis in AD mouse models.

AHN is restricted under the AD pathological conditions, which leads to a decrease in the production of newborn neurons in the hippocampus and in the plasticity of neural circuits, which ultimately makes it difficult to recover from learning/memory dysfunction in AD mice [22, 50]. The results of our team’s previous research on the treatment of AD mice with PBMT indicated that PBMT alleviated AD symptoms by attenuating AMPA receptor endocytosis [35], reducing Aβ levels [36], and promoting neurogenesis [34], but these previous work focused on direct regulation of neurons or NSCs. However, this study extended the direct processing of neurons or NSCs by PBMT to the processing of T lymphocytes. Neuroimmunomodulation is also considered as a potential treatment for AD. Some studies have shown that CD4^+^ T cells are associated with the clearance of Aβ plaques in AD pathology [51], alleviating the pathological process of AD [14, 15], and improving the cognitive ability of mice [52, 53]. The discovery of meningeal lymphatic vessels [54–56] also provides a certain feasibility basis for our research. Therefore, we shifted PBMT-treated neurons or NSCs to PBMT-treated immune cells, improving the function of immune cells to ameliorate the brain microenvironment and promote AHN in the AD model. Indeed, our results showed that PBMT promoted the secretion of cytokine IFN-γ/IL-10 from CD4^+^ T cells to positively regulate AHN. However, in addition to the changes in the function of CD4^+^ T cells after PBMT treatment of lymph nodes, some other immune cells may also contribute to this process, for example, the effect of PBMT treatment of lymph nodes on B cells requires further discussion and research. In the present study, we mainly investigated that PBMT treatment of lymph nodes can exert beneficial neurogenesis modulating effects by regulating the function of non-parenchymal CD4^+^ T cells, induction of improvement of brain microenvironment and alleviation of cognitive deficits in APP/PS1 and 3xTg-AD mouse models. However, the detailed process of how the functionally improved non-parenchymal CD4^+^ T cells reach the brain step by step after PBMT treatment, and whether some morphological or other protein expression level changes of CD4^+^ T cells occur during this process remain to be explored in depth. Our findings also suggested that IFN-γ/IL-10 protein expression was upregulated in brain tissue after PBMT treatment of lymph nodes, resulting in altered brain microenvironment, which promoted adult hippocampal neurogenesis and alleviated cognitive deficits in APP/PS1 and 3xTg-AD mice. It is well known that in addition to the improvement of the microenvironment in the neurogenic niche, the connection and feedback regulation between astrocytes, microglia, neurons, and neural stem cells are essential during neurogenesis [57–59]. This study focused on the improvement of the microenvironment of the neurogenic niche in APP/PS1 and 3xTg-AD mice, but the role of the crosstalk between neuronal/glial cells and neural stem cells on neurogenesis was less explored. Detailed studies of the process by which non-parenchymal CD4^+^ T cells with improved function after PBMT treatment of lymph nodes reach the brain and the research on the crosstalk between neural/glial cells and neural stem cells after PBMT treatment are the current focus of our team.

AHN is an important basis for maintaining the cognitive function of the brain; chronic inflammation of the peripheral or CNS inhibits AHN [60]. Neuroinflammation is a double-edged sword in the pathological development of AD. In the early stage of AD pathological development, microglia can inhibit the progression of inflammation, phagocytose Aβ plaques, and release neuroprotective factors to improve the brain microenvironment [48], which is beneficial to AHN. However, as the pathological process of AD deteriorates, microglia will be affected by inflammatory factors to aggravate the inflammatory response, and interact with astrocytes to further aggravate neurotoxicity [61, 62]; this situation is very unfavorable for AHN. Based on the above reasons, we should not only consider the influence of different stages of AD on AHN, but also consider the impact of neuroinflammation that may exist in the brain at different stages of AD on AHN. Therefore, considering the effects of the different stages of AD and the corresponding physiological conditions on AHN, this study was carried out in the early stage of AD pathology, and it aimed to alleviate the inhibition of AHN in the early stage of AD by regulating the function of peripheral non-parenchymal T lymphocytes, so as to provide a feasible treatment strategy for improving the pathological symptoms of AD. Both microglia and astrocytes play an indispensable role in the pathological neuroinflammation of AD, the activation and different background of microglia will cause them to secrete different cytokines and produce different effects, such as promoting or disrupting neuron function [61]. For example, inhibiting the levels of tumor necrosis factor-α (TNF-α), IL-1β, and interleukin-6 (IL-6) in microglia will play a neuroprotective role in neurodegenerative diseases, but microglia will also release ROS, reactive nitrogen species, and cytokines, thereby inducing a cascade of neuroinflammation and promoting neuronal apoptosis [63]. Similarly, the signal transduction and cell behavior changes of astrocytes in the pathological development of AD are also associated with neuroinflammation [64], for example, the proliferation of reactive astrocytes can lead to the activation of nuclear factor kappa-B (NF-κB) signals in astrocytes, which triggers the production of nitric oxide (NO), which has a harmful effects on neurons when it is excessive [65, 66]. In addition, astrocytes and microglia secrete a variety of cytokines or inflammatory mediators to regulate the inflammation of the CNS [67], for example, astrocytes will respond to microglia-derived IL-10 to limit the expression of pro-inflammatory genes and up-regulate the expression of anti-inflammatory genes at the same time [49]. The results of our study indicated that PBMT treatment of APP/PS1 and 3xTg-AD mice's lymph nodes decreased the reactive astrogliosis, and strengthened the phagocytosis of microglia on Aβ plaques, which suggesting that PBMT reduced the neuroinflammation cascade effect in the brain tissue to a certain extent, improved the chronic inflammation of the CNS and provided favorable conditions for AHN under AD pathology. Not only for AHN, this study also showed that PBMT treatment activated the JAK2/STAT4/STAT5 signaling pathway to promote the secretion of IFN-γ/IL-10 from CD4^+^ T cells in vivo and in vitro, thereby regulating the immune function of CD4^+^ T cells. These results further indicated that PBMT might affect the immune system of the whole body by regulating immune cells, therefore, this study might also provide a potential treatment strategy for some diseases related to the immune system, such as neurodegenerative diseases, diabetes, and even some cardiovascular diseases.

## Conclusion

In the present study, we verified that PBMT treatment activated the JAK2/STAT4/STAT5 signaling pathway, which subsequently upregulated the expression of IFN-γ/IL-10 in non-parenchymal CD4^+^ T cells, resulting in improving the expression levels of TGFβ1/IGF-1/BDNF in the brain of AD to promote AHN and synaptic plasticity of newborn neurons. Furthermore, our study also demonstrated that PBMT, as a non-invasive and drug-free physiotherapy strategy, has potential therapeutic value in regulating the function of non-parenchymal immune cells to ameliorate the niche microenvironment of NSCs and attenuate cognitive deficits in APP/PS1 and 3xTg-AD mice.

## Supplementary Information

**Additional file 1: Fig. S1. **Effects of photobiomodulation therapy (PBMT)-treated lymph nodes on the expression of TGFβ1/IGF-1/BDNF, activation of microglia and dystrophic neurites in the brain tissue of APP/PS1 and 3xTg-AD mice. **(A-C).** The expression of transforming growth factor-β1 (TGFβ1) **(A)**/ insulin-like growth factors-1 (IGF-1) **(B)**/brain-derived neurotrophic factor (BDNF) **(C)** in the brain tissue were detected by flow cytometer (*n* = 3–5 per group). **(D-E).** The number of ionized calcium bindingadaptor molecule-1 (Iba-1) ^+^ cells in the brain tissue of APP/PS1 and 3xTg-AD mouse were detected **(D)** and analyzed **(E)** by flow cytometer, (*n* = 3–5 per group). **(F).** Representative images of recombinant lysosomal associated membrane protein 1 (lamp1)^+^ (dystrophic neurites staining) expression cells and amyloid-β (Aβ) (Aβ plaque staining) deposition in APP/PS1 and 3xTg-AD mouse brain at the end of PBMT, DAPI was used to stain nucleus. Scale bars, 50 μm. **(G).** Quantitative analyses of percentage of lamp1^+^ area in the APP/PS1 and 3xTg-AD mouse brain after PBMT-treated lymph nodes, (*n* = 5–7 per group). All quantifications are presented as mean ± SEM and were analyzed by One-way ANOVA test; ***p < 0.001, **p < 0.01, *p < 0.05 versus WT group; ###*p* < 0.001, ##*p* < 0.01, #*p* < 0.05 versus indicated group. **Fig. S2. **Effects of PBMT-treated APP/PS1 and 3xTg-AD mouse lymph nodes on the concentration of IFN-γ/IL-10 in serum, the number of IFN-γ^+^ IL-10^+^ T cells and IL-10^+^ CD4^+^ T cells in the spleen. **(A-B).** The concentration of IFN-γ **(A)**/IL-10 **(B)** in serum were measured by enzyme linked immunosorbent assay (ELISA) after PBMT-treating APP/PS1 and 3xTg-AD mouse lymph nodes, (*n* = 3-4 per group). **(C-F).** CD4 antibody was used to staining the CD4^+^ T cells in the spleen, and then the expression **(C)** and analyzed **(D)** of IFN-γ in CD4^+^ T cells**,** the expression **(E)** and analyzed of IL-10 **(F)** in CD4^+^ T cells were detected and analyzed by flow cytometer, (*n* = 4 per group). All quantifications are presented as mean ± SEM and were analyzed by One-way ANOVA test; ***p < 0.001, **p < 0.01, *p < 0.05 versus WT group; ###*p* < 0.001, ##*p* < 0.01 versus indicated group. **Fig. S3. **Effects of PBMT-treated lymph nodes on the activation of CD4^+^ T cells, and the expression of Tuj1 and PSD95 in the brain tissue of APP/PS1 and 3xTg-AD mice. **(A).** The number of CD4^+^ CD69^+^ T cells in the brain tissue of six groups were analyzed by flow cytometry, (*n* = 5-6 per group). The representative images of **(A)** were provided in Fig. 4F. **(B-C).** Western blotting analysis **(B)** and quantification **(C)** of Tuj1 and PSD95 protein expression in APP/PS1 and 3xTg-AD mouse brain after PBMT-treated lymph nodes, (*n* = 4 per group). All quantifications are presented as mean ± SEM and were analyzed by One-way ANOVA test; ***p < 0.001, *p < 0.05 versus WT group; ###*p* < 0.001, ##*p* < 0.01, #*p* < 0.05 versus indicated group. **Tables S1** Laser parameters used in vivo*.* **Tables S2** Laser parameters used in vitro.

## Data Availability

All raw data used in this manuscript are available on reasonable request.

## Abbreviations

PBMT: Photobiomodulation therapy
AD: Alzheimer's disease
AHN: Adult hippocampal neurogenesis
APP/PS1: Amyloid precursor protein/presenilin 1
3xTg-AD: 3xTg (APP/PS1/tau)-AD
WT: Wild-type
Aβ: Amyloid-β
TGFβ1: Transforming growth factor-β1
NSCs: Neural stem cells
ROS: Reactive oxygen species
CNS: Central nervous system
Tuj1: Neuronal class-III β-tubulin
GFAP: Glial fibrillary acidic protein
SGZ: Subgranular zone
NFTs: Neurofibrillary tangles
PSD95: Postsynaptic density protein 95
NAC: N-Acetyl cysteine
MWM: Morris water maze
CcO: Cytochrome c oxidase
AMPARs: α-Amino-3-hydroxy-5-methyl-4-isoxazole-propionic acid receptors
IL-1β: Interleukin-1β
TNF-α: Tumor necrosis factor-α
IL-6: Interleukin-6
NF-κB: Nuclear factor kappa-B
NO: Nitric oxide
CM: Conditioned medium
Lamp1: Recombinant lysosomal associated membrane protein 1
Iba-1: Ionized calcium binding adaptor molecule-1
BSA: Bovine serum albumin
OCT: Optimal cutting temperature
PFA: Paraformaldehyde
IFN-γ: Interferon-γ
IL-10: Interleukin-10
JAK2: Janus kinase 2
STATs: Signal transducer and activator of transcriptions
IGF-1: Insulin-like growth factors-1
BDNF: Brain-derived neurotrophic factor
MHC: Major histocompatibility complex
BBB: Blood–brain barrier
CP: Choroid plexus
CSF: Cerebrospinal fluid
