# Supplementary material for: Promoted CD4+ T cell-derived IFN-γ/IL-10 by photobiomodulation therapy modulates neurogenesis to ameliorate cognitive deficits in APP/PS1 and 3xTg-AD mice
Source: J Neuroinflammation. 2022 Oct 10;19:253. doi: 10.1186/s12974-022-02617-5 (PMC9549637; doi:10.1186/s12974-022-02617-5)
Supplement: Supplementary file 1 — Additional file 1: Fig. S1. Effects of photobiomodulation therapy (PBMT)-treated lymph nodes on the expression of TGFβ1/IGF-1/BDNF, activation of microglia and dystrophic neurites in the brain tissue of APP/PS1 and 3xTg-AD mice. (A-C). The expression of transforming growth factor-β1 (TGFβ1) (A)/ insulin-like growth factors-1 (IGF-1) (B)/brain-derived neurotrophic factor (BDNF) (C) in the brain tissue were detected by flow cytometer (n = 3–5 per group). (D-E). The number of ionized calcium bindingadaptor molecule-1 (Iba-1) + cells in the brain tissue of APP/PS1 and 3xTg-AD mouse were detected (D) and analyzed (E) by flow cytometer, (n = 3–5 per group). (F). Representative images of recombinant lysosomal associated membrane protein 1 (lamp1)+ (dystrophic neurites staining) expression cells and amyloid-β (Aβ) (Aβ plaque staining) deposition in APP/PS1 and 3xTg-AD mouse brain at the end of PBMT, DAPI was used to stain nucleus. Scale bars, 50 μm. (G). Quantitative analyses of percentage of lamp1+ area in the APP/PS1 and 3xTg-AD mouse brain after PBMT-treated lymph nodes, (n = 5–7 per group). All quantifications are presented as mean ± SEM and were analyzed by One-way ANOVA test; ***p < 0.001, **p < 0.01, *p < 0.05 versus WT group; ###p < 0.001, ##p < 0.01, #p < 0.05 versus indicated group. Fig. S2. Effects of PBMT-treated APP/PS1 and 3xTg-AD mouse lymph nodes on the concentration of IFN-γ/IL-10 in serum, the number of IFN-γ+ IL-10+ T cells and IL-10+ CD4+ T cells in the spleen. (A-B). The concentration of IFN-γ (A)/IL-10 (B) in serum were measured by enzyme linked immunosorbent assay (ELISA) after PBMT-treating APP/PS1 and 3xTg-AD mouse lymph nodes, (n = 3-4 per group). (C-F). CD4 antibody was used to staining the CD4+ T cells in the spleen, and then the expression (C) and analyzed (D) of IFN-γ in CD4+ T cells, the expression (E) and analyzed of IL-10 (F) in CD4+ T cells were detected and analyzed by flow cytometer, (n = 4 per group). All quantifications are presented as [file 12974_2022_2617_MOESM1_ESM.doc]

**Additional file 1: figures and tables**

**Supplemental figures**


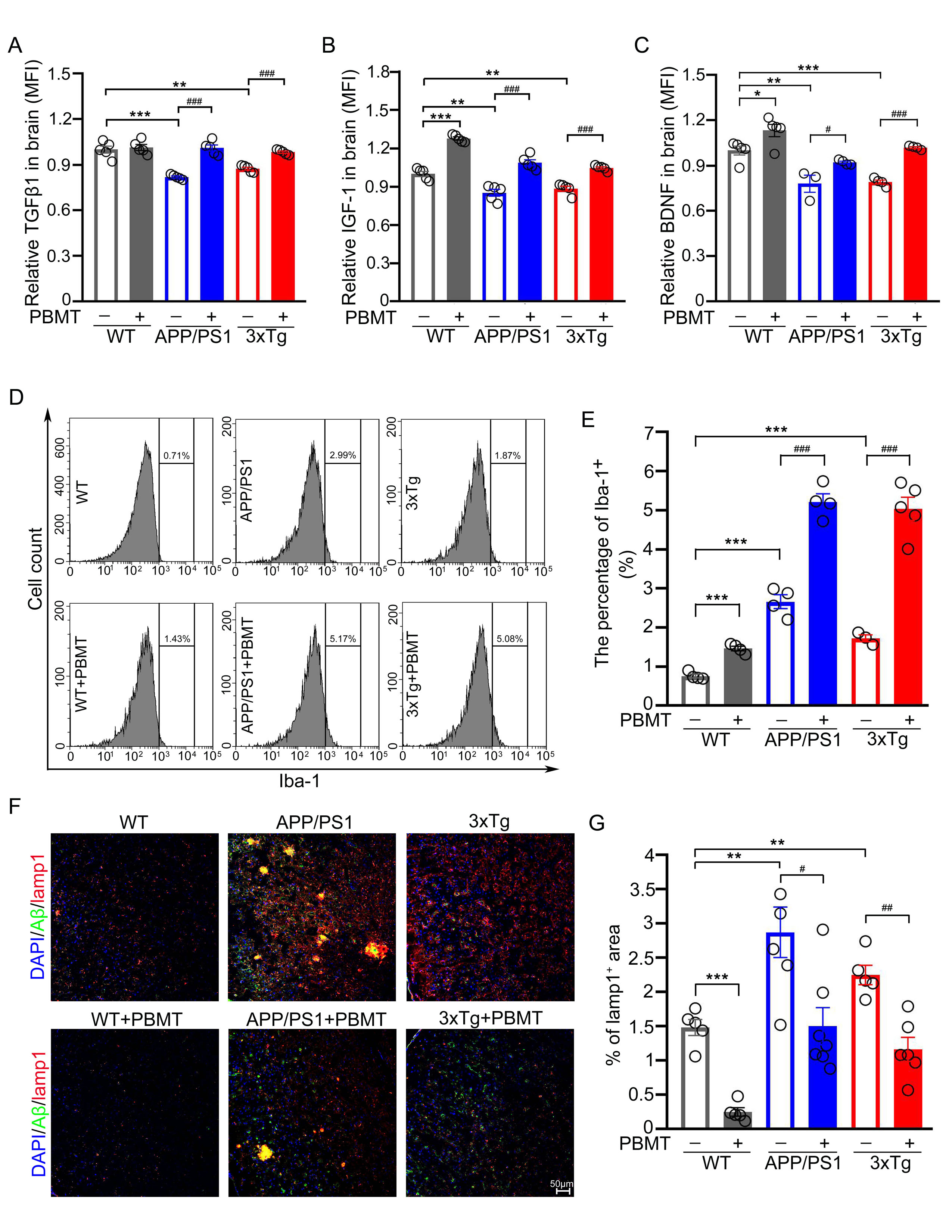


**Fig. S1. Effects of photobiomodulation therapy (PBMT)-treated lymph nodes on the expression of TGFβ1/IGF-1/BDNF, activation of microglia and dystrophic neurites in the brain tissue of APP/PS1 and 3xTg-AD mice**

**(A-C).** The expression of transforming growth factor-β1 (TGFβ1) **(A)**/ insulin-like growth factors-1 (IGF-1) **(B)**/ brain-derived neurotrophic factor (BDNF) **(C)** in the brain tissue were detected by flow cytometer (*n* = 3-5 per group). **(D-E).** The number of ionized calcium bindingadaptor molecule-1 (Iba-1) ^+^ cells in the brain tissue of APP/PS1 and 3xTg-AD mouse were detected **(D)** and analyzed **(E)** by flow cytometer, (*n* = 3-5 per group). **(F).** Representative images of recombinant lysosomal associated membrane protein 1 (lamp1)^+^ (dystrophic neurites staining) expression cells and amyloid-β (Aβ) (Aβ plaque staining) deposition in APP/PS1 and 3xTg-AD mouse brain at the end of PBMT, DAPI was used to stain nucleus. Scale bars, 50 μm. **(G).** Quantitative analyses of percentage of lamp1^+^ area in the APP/PS1 and 3xTg-AD mouse brain after PBMT treated lymph nodes, (*n* = 5-7 per group). All quantifications are presented as mean ± SEM and were analyzed by One-way ANOVA test; ****p* < 0.001, ***p* < 0.01, **p* < 0.05 versus WT group; ###*p* < 0.001, ##*p* < 0.01, #*p* < 0.05 versus indicated group.


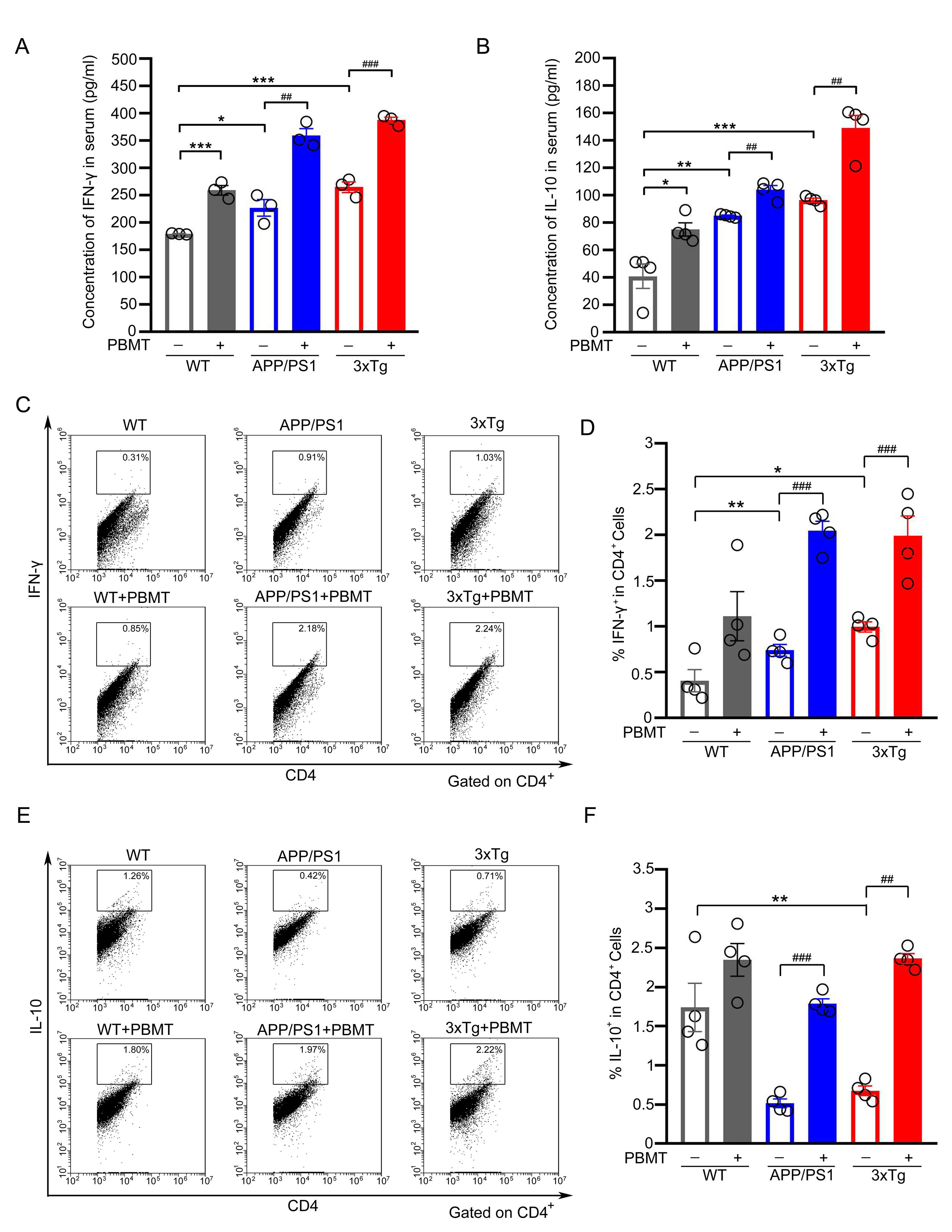


**Fig. S2. Effects of PBMT-treated APP/PS1 and 3xTg-AD mouse lymph nodes on the concentration of IFN-γ/IL-10 in serum, the number of IFN-γ^+^ IL-10^+^ T cells and IL-10^+^ CD4^+^ T cells in the spleen**

**(A-B).** The concentration of IFN-γ **(A)**/IL-10 **(B)** in serum were measured by enzyme linked immunosorbent assay (ELISA) after PBMT-treating APP/PS1 and 3xTg-AD mouse lymph nodes, (*n* = 3-4 per group). **(C-F).** CD4 antibody was used to staining the CD4^+^ T cells in the spleen, and then the expression **(C)** and analyzed **(D)** of IFN-γ in CD4^+^ T cells**,** the expression **(E)** and analyzed of IL-10 **(F)** in CD4^+^ T cells were detected and analyzed by flow cytometer, (*n* = 4 per group). All quantifications are presented as mean ± SEM and were analyzed by One-way ANOVA test; ****p* < 0.001, ***p* < 0.01, **p* < 0.05 versus WT group; ###*p* < 0.001, ##*p* < 0.01 versus indicated group.


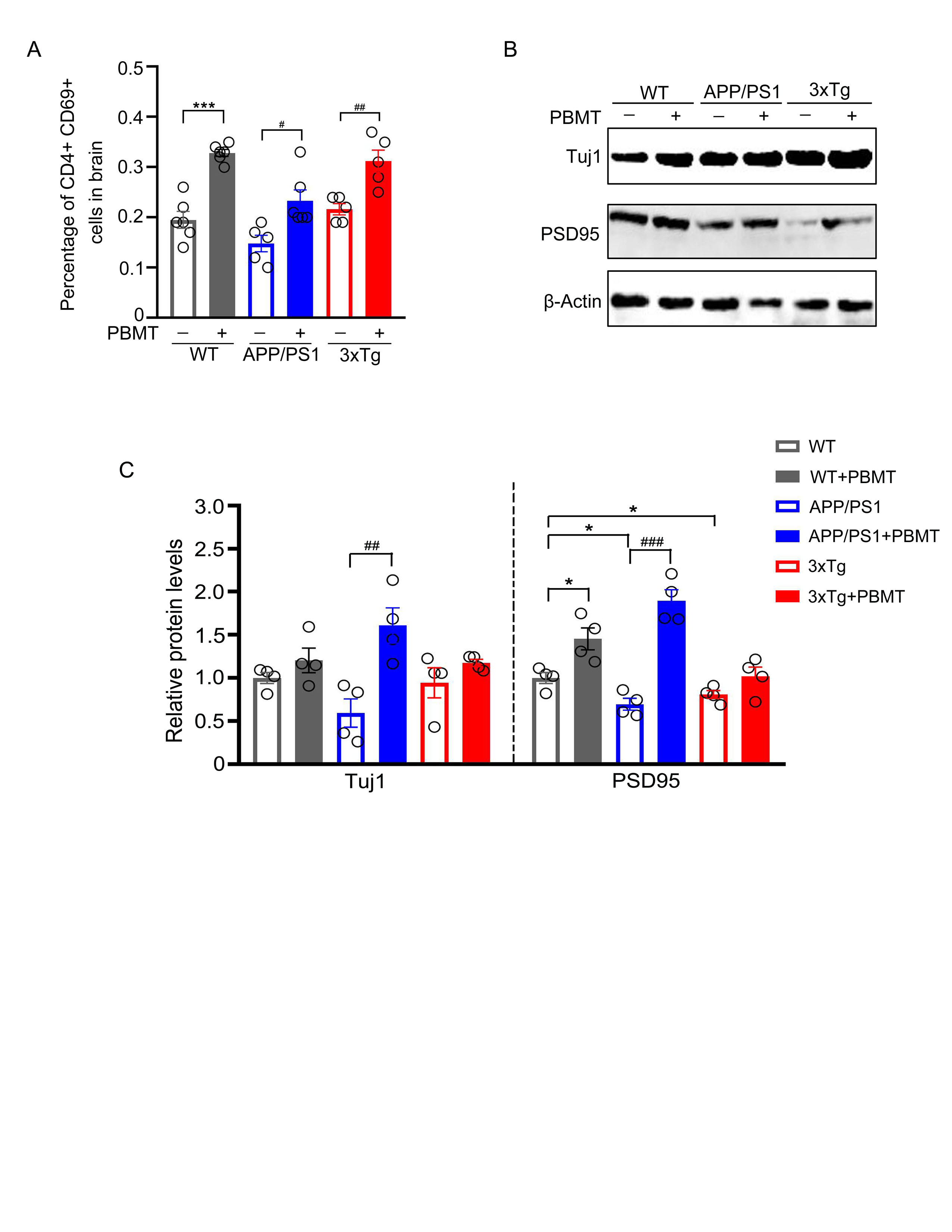


**Fig. S3. Effects of PBMT-treated lymph nodes on the activation of CD4^+^ T cells, and the expression of Tuj1 and PSD95 in the brain tissue of APP/PS1 and 3xTg-AD mice**

**(A).** The number of CD4^+^ CD69^+^ T cells in the brain tissue of six groups were analyzed by flow cytometry, (*n* = 5-6 per group). The representative images of **(A)** were provided in Fig. 4F. **(B-C).** Western blotting analysis **(B)** and quantification **(C)** of Tuj1 and PSD95 protein expression in APP/PS1 and 3xTg-AD mouse brain after PBMT-treated lymph nodes, (*n* = 4 per group). All quantifications are presented as mean ± SEM and were analyzed by One-way ANOVA test; ****p* < 0.001, **p* < 0.05 versus WT group; ###*p* < 0.001, ##*p* < 0.01, #*p* < 0.05 versus indicated group.

**Tables S1** laser parameters used *in vivo*

| Light source Semiconductor laser | Semiconductor laser |
| --- | --- |
| Beam spot size at target (cm^2^) | 7.06 cm^2^ |
| Irradiation time (sec) | 600 sec |
| Power density over the mice epidermis (mW/mm^2^) | 0.1667 mW/mm^2^ |
| Power density reaching the mice lymph nodes (mW/mm^2^) | 0. 0333 mW/mm^2^ |
| Energy density in the mice lymph nodes (J/cm^2^) | 2 J/cm^2^ |
| Energy density over the mice epidermis (J/cm^2^) | 10 J/cm^2^ |
| Frequency of treatment sessions | Once a day |
| Emission | Continuous wave |

**Tables S2** laser parameters used *in vitro*

| Light source Semiconductor laser | Semiconductor laser |
| --- | --- |
| Beam spot size at target (cm^2^) | 9.6 cm^2^ |
| Irradiation time (sec) | 300 sec |
| Power density over the cells (mW/mm^2^) | 0.0667 mW/mm^2^ |
| Energy density over the cells (J/cm^2^) | 2 J/cm^2^ |
| Emission | Continuous wave |
